# Proprioceptive manipulations in orthograde posture modulate postural control in low back pain patients: a pilot study

**DOI:** 10.1038/s41598-022-10701-2

**Published:** 2022-04-27

**Authors:** Nicolas Bouisset, Augustin Roland-Gosselin, Maurice Ouaknine, Jean Luc Safin

**Affiliations:** 1 Department of Kinesiology, Western University, London, ON N6A 3K7 Canada; 2 GRETM/Groupe de Recherche et d’Enseignement en Thérapie Manuelle, 03700 Bellerive-sur-Allier, France; 3 Université de Grenoble-Alpes, Laboratoire AGIM, 38700 La Tronche, France; 4 Institut de Formation en Masso-Kinésithérapie, 03200 Vichy, France

**Keywords:** Neuroscience, Rehabilitation, Musculoskeletal system

## Abstract

As we stand upright, perceptual afferences are crucial to successfully help generating postural motor commands. Non-Specific Low Back Pain patients frequently demonstrate a lack of proprioceptive acuity, often translating into postural control deficiencies. For the first time, to our knowledge, we studied the postural effects of proprioceptive manipulations in orthograde posture on Non-Specific Low Back Pain patients. Using static posturography recordings, we computed sway speed, speed variance, and the main direction of sway. We also addressed the patient’s subjective feedbacks after being manipulated. Five minutes after the proprioceptive manipulations, our results revealed decreased speed and speed variance outcomes, but the main direction of sway was not modulated. Furthermore, after the proprioceptive manipulations, the patients also self-reported improved clinical outcomes. These findings provide new knowledge opening new fields of research as well as potential treatment strategies in Low Back Pain patients.

## Introduction

Human motor behavior depends on sensory inputs processing and motor outputs. Sensory acuity is primordial to execute precise motor tasks, and motor signals impact sensory integration while voluntarily moving^1^. Humans navigate and act upon their environment upright. Therefore, orthograde posture is the primary interface between perception and action. Postural control is the foundation on which general human motor control is based^2^, as balance needs to be dynamically adjusted for movements to be precise and accurate. Furthermore, erect posture also serves as a fundamental reference frame around which the motor output is controlled and organized^2^. Indeed, the control of axial and proximal musculature provides stabilizing support for increased distal motor skill performances^3^. Moreover, as we move, the ongoing changes in the body’s geometry generate dynamical shifting of the center of mass. Such changes need pre-established modulatory control by Anticipatory Postural Adjustments (APAs)^4^, to avoid instabilities or falls^5^. Such adjustments derive from cortical internal models regularly updated through multisensory integration processes where vision, somatosensory, proprioception as well as vestibular information are bound within the central nervous system^6,7^.

People experiencing Low Back Pain demonstrate motor control changes^8^. Non-Specific Low Back Pain (NSLBP) patients, for instance, often demonstrate postural control deficiencies^9^. Non-Specific Low Back Pain is defined as “pain that cannot be attributed to a recognizable pathology, (e.g., infection, tumor, osteoporosis, fracture, structural deformity, inflammatory disorders, radicular syndrome, or cauda equina syndrome)”^10^, and much debate is still ongoing on how to treat such an issue given the various approaches used clinically^11^.

Acute NSLBP modifies the way proprioceptive cues are integrated. Patients with NSLBP have less lower-back proprioceptive acuity and awareness^12^. Moreover, with NSLBP, proprioception is reweighted in such a way that the ankle information gain increases while the lumbar information gain decreases accordingly^13,14^. Altogether, these weighting mechanisms modulate integrative processes inducing plastic changes not only at the dorsal horn of the spinal cord but also at the cortical level^15^.

If maintained, such modulatory processes could have important consequences. First, because of the reweighting proprioceptive mechanisms, the wrongly modulated internal models could impact the APAs. With altered postural control, NSLBP patients could be more prone to instabilities and falls^16^. Second, the reweighting mechanisms implicated in the emergence of NSLBP could, through the neurological plastic changes, help to transition from acute pain states to more chronic ones^15^.

In this context, facilitating proprioceptive integration and improving postural control in NSLBP patients could not only be potentially beneficial for their recovery but could also avoid patients from evolving towards more chronic painful states.

A French manual therapy system claims to modulate motor control in general and postural control more specifically^17^. Intriguingly, such a system uses proprioceptive manipulative techniques while the patient stands erect in their functional orthograde posture. Yet, to our knowledge, such a system has never been scientifically evaluated and thus published in an international peer-reviewed journal. No formal physiotherapy-specific graduate programs (i.e., masters and PhDs) are available in France. This could potentially explain why such a system was left unresearched to this day, even though thousands of French physiotherapists use it daily in their practice. Thus, for the first time, to our knowledge, the study described in this paper investigated whether such manipulations modulate postural control in NSLBP patients.

The aim of this study was twofold. First, the study was meant to investigate whether the known altered postural control in LBP patients^8,9^ could be modulated with such manipulations. Second, we also wanted to address how the patients subjectively felt after they had been manipulated. Given these proprioceptive manipulations are used as treatment techniques in France, we hypothesized (1) improved postural control and (2) that the patients would report positive feedback about how they felt after the manipulations.

## Methods

### Participants

After a referral from their primary physician, we asked patients with an NSLBP medical diagnosis if they would be willing to participate in the current study. Men and women aged 20 to 60 years diagnosed with acute (less than 3 months) NSLBP with or without leg pain were recruited for the study. To avoid any preconceived bias, we excluded patients who had already been treated with the techniques evaluated in the current work. In cooperation with the patients’ general practitioner and based on their medical record, exclusion criteria were set as follows: (1) history of lumbar surgery, known rheumatic, neurologic, inflammatory disorder, and mental disease, (2) pregnancy or suspicion of pregnancy, (3) suspicion of infection, malignancy, osteoporosis, fracture, (4) patients with any sort of medication, (5) known vestibular and visual diseases, and (6) overt loss of somatic sensation.

The sample size was calculated using the PWR package in R. Assuming two-tailed testing, a level of significance of α = 0.05, a power of 0.8, and a value of d set at 0.8, a minimum of 33 participants was needed. Given these criteria, forty-two patients were recruited for the study (see Table 1 for descriptive statistics).

**Table 1:** Descriptive statistics for the participants. Mean and standard deviations values for age, height, weight, and shoe size.

	Age	Height (cm)	Weight (cm)	Shoe size (European)
Female (n = 24)	39 ± 13	169 ± 7	65 ± 6	38 ± 2
Male (n = 18)	36 ± 9	175 ± 10	76 ± 9	42 ± 2

### Procedure

The experiment consisted of a single session lasting about half an hour during which the manipulations were applied once to each body part. During the visit, two postural control measurements were done. Following a repeated-measures plan, the first recording was done before the proprioceptive manipulations and the second one was executed 5 min after.

**Figure 1:**
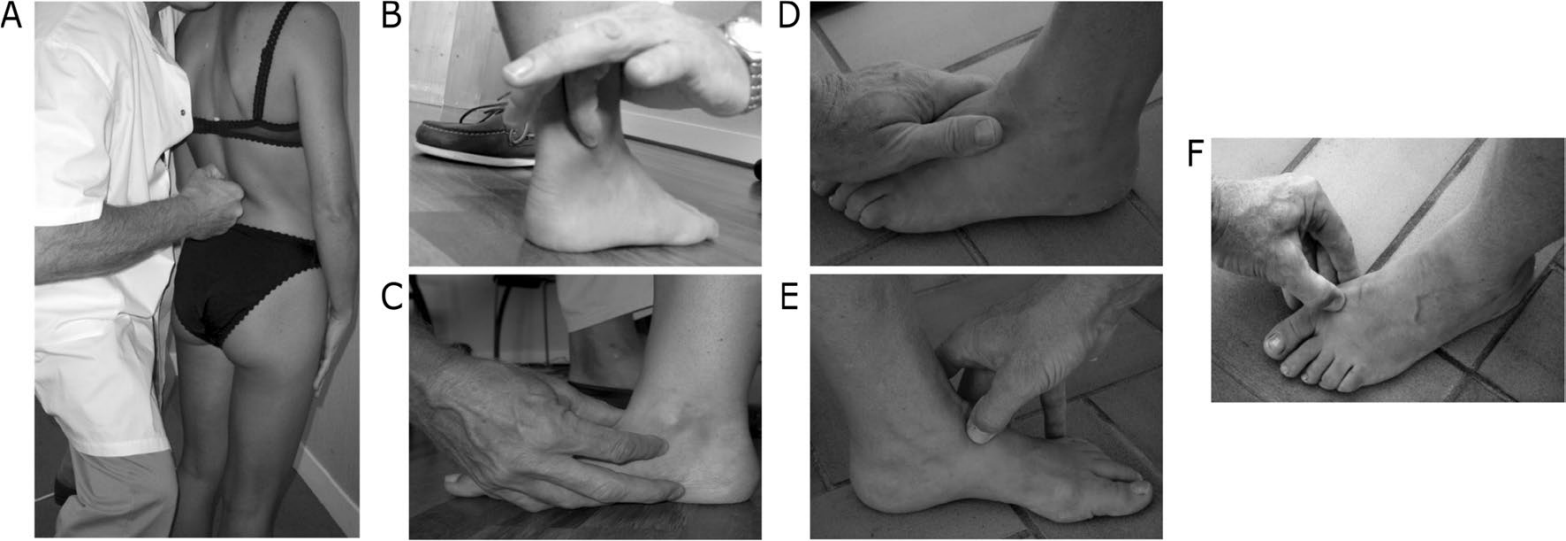
Lumbar, ankles and feet manipulation techniques.

These techniques are always executed upright, in the patient’s functional orthograde posture. Traditionally, whenever a musculoskeletal problem is treated using these proprioceptive techniques, the ankles and feet are always manipulated. Because the proprioceptive gain is favorably reweighted at the ankles in NSLBP patients^13,14^, we decided to incorporate ankles and feet manipulations and follow tradition. Therefore, we executed the manual techniques once over each functional anatomical target (Fig. 1). We manipulated every level of the lumbar spine and both legs at the ankles and feet. Each manipulation technique lasted approximately 1 to 2 s.

Each spinal process from L1 to L5 is manipulated following the same technique (Fig. 1A). This technique is done with the proximal interphalangeal joint of the index finger by linearly crossing the spinal processes from one side to the other. In this concept, and contrary to most manual manipulation techniques, the actual orientation of the technique is always the same depending on handedness. Indeed, the orientation of the technique is inverted whether the therapist is right or left-handed. For a right-handed therapist (which is the case in this study) the lumbar techniques are always oriented from right to left and anteriorly to the spinal process.

Both fibularis longus and fibularis brevis muscles are alternatively manipulated slightly above the posterior aspect of the lateral malleolus (Fig. 1B). The technique is done by pulling the fibularis tendons forwards with the medial border of the index finger of the ipsilateral hand (i.e., right hand for the left foot and vice versa). Regarding the tibialis posterior muscles, the technique is executed by pulling the tendon forward and up with the pulp of the index finger of the contralateral hand (Fig. 1C). When manipulating the tibialis anterior muscles, the technique is executed with the pulp of the thumb of the contralateral hand pushing forward and laterally (Fig. 1D)**.**

Concerning the extensor digitorum muscles, the technique is executed with the medial interphalangeal border of the thumb of the contralateral hand pushing over the tendons upward and medially (Fig. 1E). Finally, the extensor hallucis longus are both manipulated by pushing the tendon medially with the thumb of the contralateral hand (Fig. 1F).

**Figure 2:**
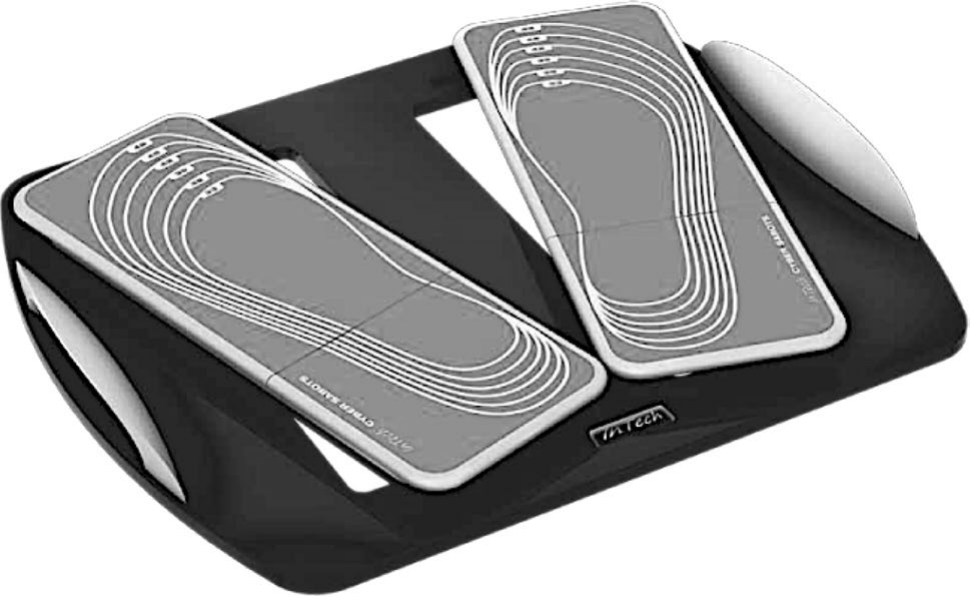
Two distinct synchronized patented dynamometric platforms, one for each foot, constitute the Cyber-Sabots. The silk-screened markings of each foot sole upon the two mono plates enable proper placement according to the patient feet size, maximizing standardization.

A force plate (Fig. 2) (Cyber-Sabots, Innovative Technology, France) was used to collect participants’ body sway at 40 Hz. The Center of Pressure (COP) variables were calculated post-recording (see “Data analysis”). All data were acquired in a noise attenuated environment with a fixed temperature (i.e., 21 °C).

**Figure 3:**
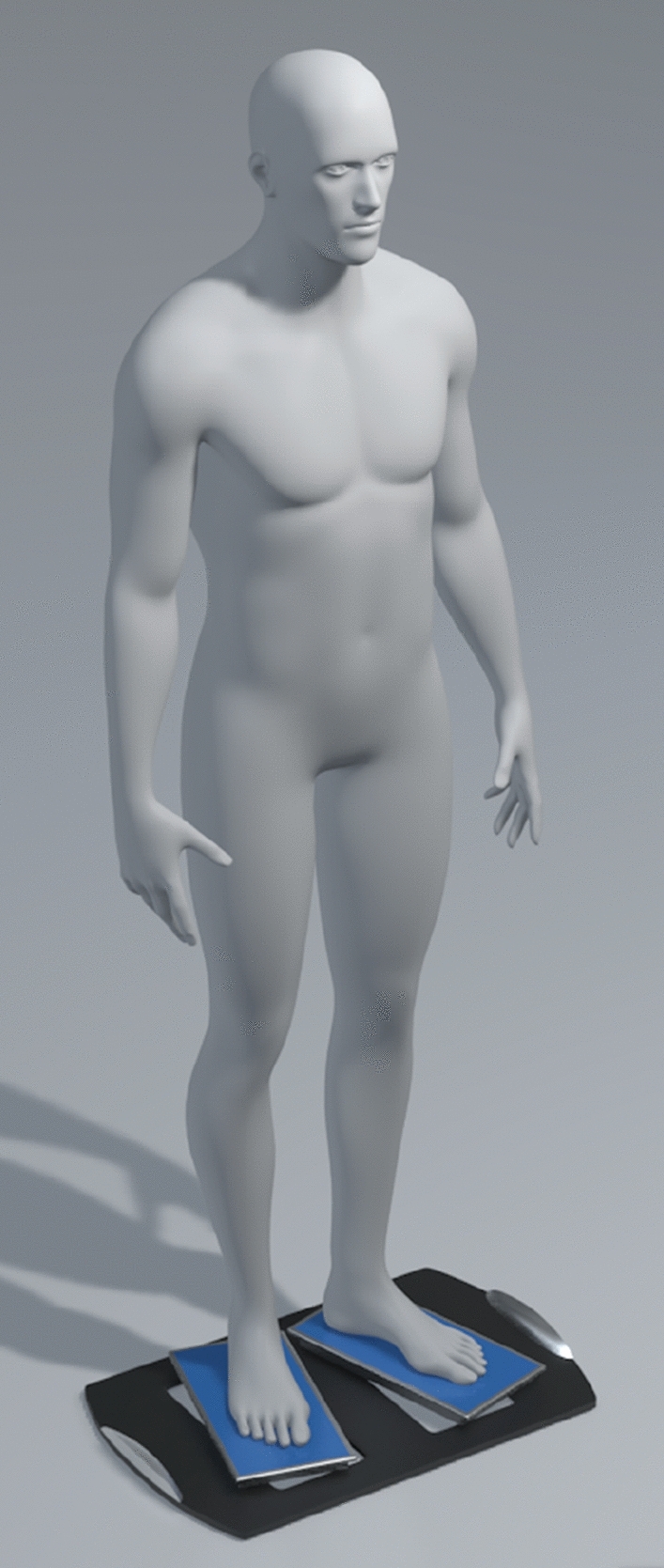
Body position during the postural control measurements. Volunteers stood eyes opened, arms by their side and feet positioned at a 30° angle, specifically located following the landmarks engraved on the platforms.

Given higher consistency in COP measurements, the participants were explicitly asked to “stand as still as possible”^18^, on the force plate with their eyes open, arms along the sides (Fig. 3), and feet at an angle of 30° (Figs. 2 and 3). The feet position was also achieved by following the landmarks on the platforms relative to their shoe size (Fig. 2). The feet position was also chosen to maximize measurements standardization. Participants stood barefoot.

After obtaining the baseline recording, the physiotherapist immediately manipulated the patients. Five minutes of rest were given between the end of the manipulation treatment and the second postural control measurement. During the resting periods, participants could relax but could not sit to avoid any cerebrovascular alterations that could also bias postural outcomes after standing back up^19^. Each recording lasted for 51.2 s.

Finally, we requested the patients’ feedback concerning what they felt during, as well as after the manipulations and at the end of the resting period. Right after the manipulations, the physiotherapist asked the following questions: (1) “Would you mind telling us what you have specifically experienced during the manipulations?” and (2) “Is there anything about the treatment you would like to tell us right now?”. Then, right before the second recording, the physiotherapist asked: “Is there anything you would like to tell us about the treatment before we proceed to the second measurement?”. Finally, the physiotherapy asked this last question: “Is there anything you would like to tell us about the treatment before we end this session?”.

The study was conducted in compliance with the protocol “Good Clinical Practices” and the Declaration of Helsinki principles. In accordance with French law, all participants gave their verbal and written consent to participate after being informed about the study protocol, and their confidentiality and anonymity were always preserved. The participation in the study did not modify usual rehabilitation.

### Data analysis

**Figure 4:**
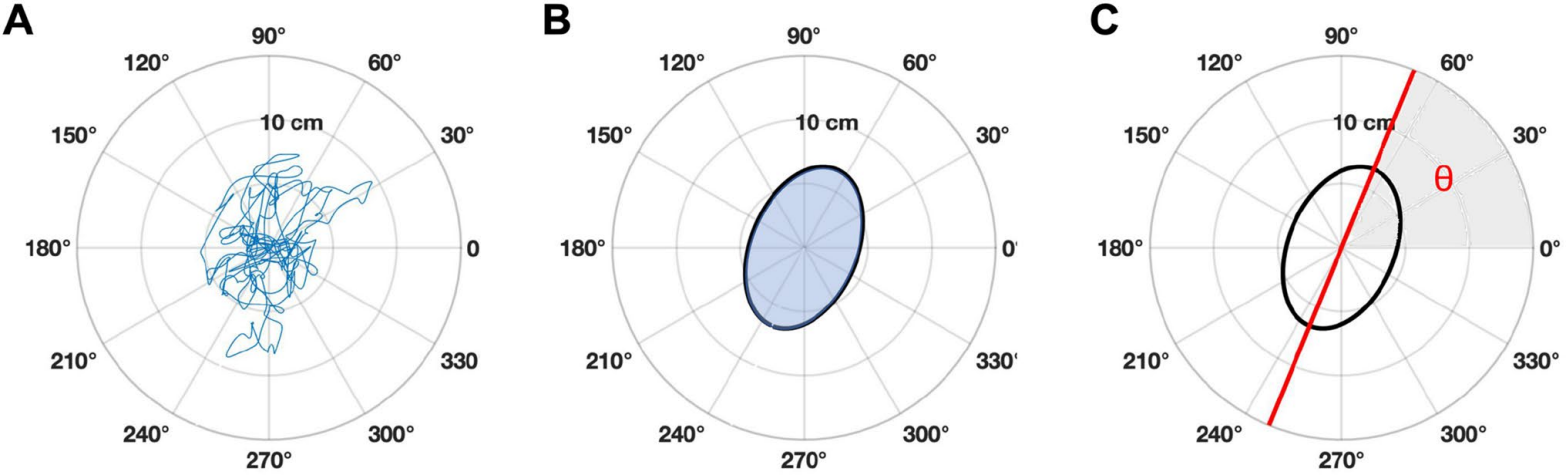
Graphical representation of the dependent variables. In (**A**) the movement of COP is represented by the blue line. The total length of the blue line divided by the time spent to travel this distance provides the speed variable. In (**B**) the movement of the COP can be summarized by the blue shaded area representing the 95% confidence interval ellipse area. The major axis of the ellipse is used to compute the main direction of sway. In (**C**) The red line represents the main direction of sway at an angle θ, symbolized by the grey shaded area. A direction of sway at 90 degrees angle would indicate a pure antero-posterior sway.

The COP time-series were filtered with a low-pass bidirectional 4th order Butterworth zero-phase digital filter with a cut-off frequency of 8 Hz. As advised by Winter^20^, the cut-off frequency was determined with a residual analysis, which was done using a customized Matlab program. Sway characteristics were also computed using a customized Matlab program. Classically sway variables are analyzed on orthogonal antero-posterior (AP) and medio-lateral (ML) axes independently. However, AP-ML analyses are known to be biased by biomechanical factors^21^ such as foot positions and the patients’ feet were constrained to a 30° angle. Secondly, AP and ML data are not independent as balance is controlled by coordinating the body in space in both dimensions simultaneously^22^. Therefore, we favored planar sway analyses over AP-ML one-dimensional analyses. Among classical sway variables, the path length (the total length of COP excursion) is reported as the more sensitive and reliable outcome^23,24^. Thus, we computed path length as the total sum of the distances between each point in the AP-ML plane (Fig. 4A). However, because path length varies with recording data time, it is often hard to compare results from one study to another. Therefore, mean velocity called speed herein (path length over time) was retained.

Also, the speed variance of the COP was found to be highly correlated with the electromyographic activity of the postural muscles^25,26^. Thus, to provide an estimation of the energy spent to stabilize one’s posture, we computed speed variance as $\frac{{\sum (x-\overline{x })}^{2}}{N-1}$, where x is the instantaneous velocity,$\overline{x }$ is the mean velocity during the trial, and N the number of points in the time series.

Finally, we investigated whether the manipulations would modify the orientation of sway in space. Thus, we analyzed the main direction of sway which was computed as the major axis of the 95% confidence interval ellipse area (Fig. 4B,C). To compute the sway area for each trial, we conducted a Principal Component Analysis (PCA) on COP datasets as prescribed by Oliveira et al.^27^. The main direction of sway is described by the first Principal Component (PC1) accounting for the largest part of the COP time-series’ variance. θ, the angle between the ML axis and the PC1 axis was computed to describe the main direction of sway (Fig. 4C). θ was always presented within 0° and 180° regardless of the direction of the movement towards the right or the left: 0° being aligned with the ML axis toward the right side of the participant.

### Statistical analysis

We performed all linear statistical analyses using R version 3.3.2^28^ and all circular statistics using the CircStat toolbox in Matlab^29^. A level of significance of α = 0.05 was adopted throughout data analysis. To investigate postural control before and after the manipulations, given our repeated-measures plan, we implemented paired-samples t-tests to analyze the impact on speed and speed variance. For θ analyses, we first ensured that θ data samples were not distributed uniformly, using Rao’s spacing test for circular uniformity. Mean θ and Angular Deviation (± AD) were used to describe the main direction of sway. A Watson-Williams two-sample test was used to investigate the effect of the manipulations on the direction of the sway^29^.

## Results

### Effects of proprioceptive manipulations on postural control

**Figure 5:**
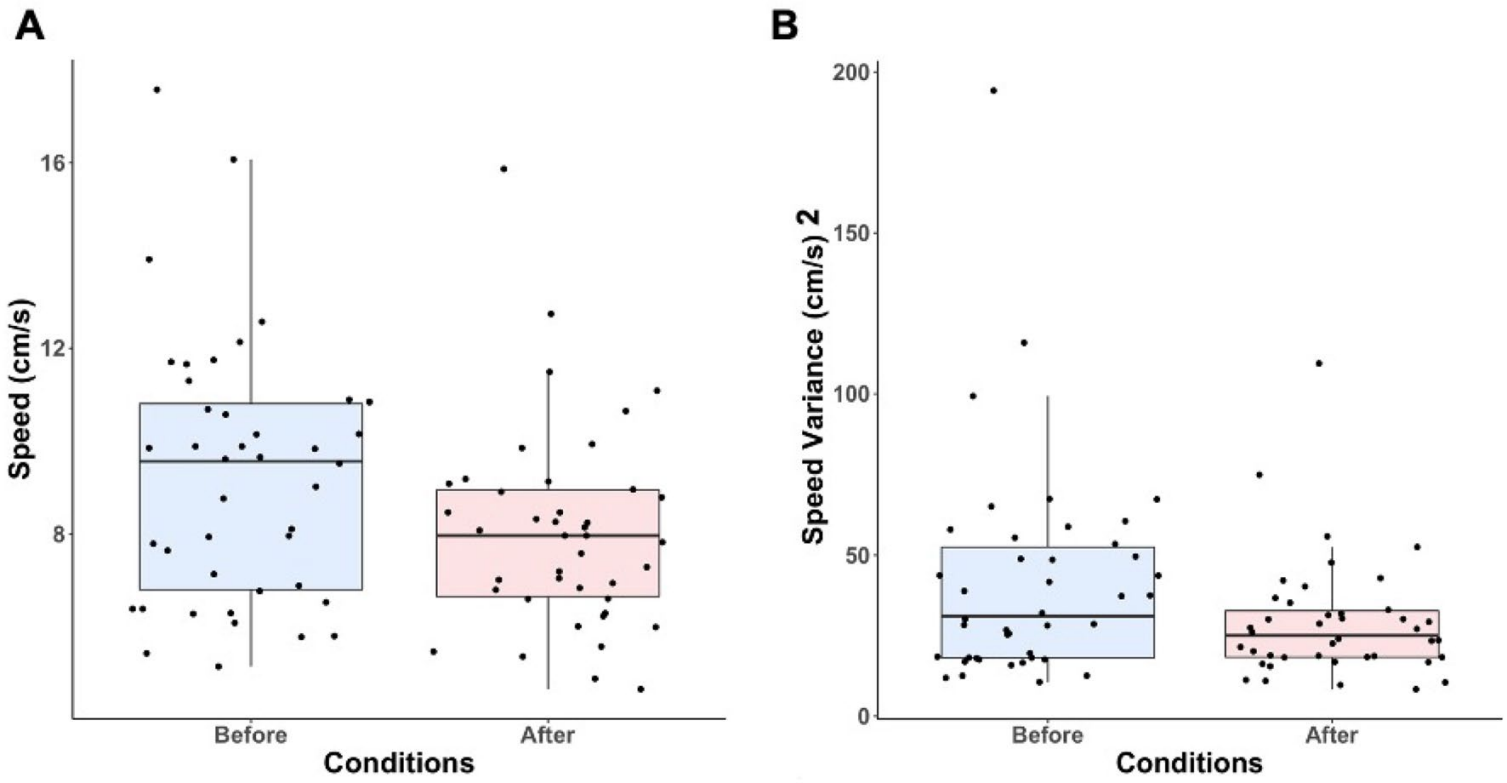
Box plots for speed (**A**) and speed variance (**B**) for before vs after manipulations.

**Figure 6:**
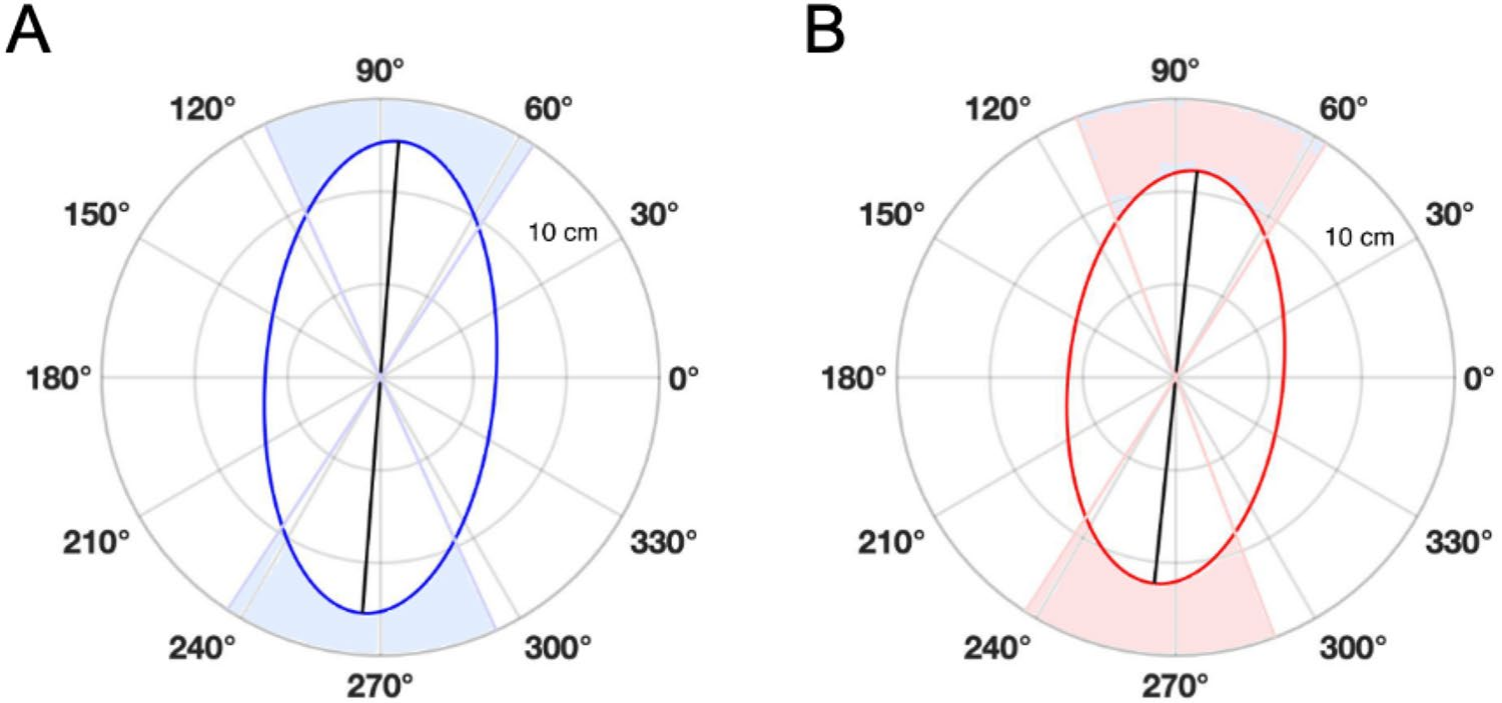
Mean direction of sway for before (**A**) vs after (**B**) manipulations. The black lines represent the main direction of sway (PC1) at the angle θ. The length of each black line is proportional to the mean quantity of movement expressed by the participants. Ellipses are a representation of the mean 95% area of COP displacement. Light blue (Before) and light red (After) shaded areas represent the angular deviations for each condition.

Speed (t (41) = 4.285, p = 0.0001, R^2^ = 0.31) (Fig. 5A) and the speed variance (t (41) = 3.094, p = 0.0035, R^2^ = 0.19) (Fig. 5B) were both significantly decreased after the manipulations. Both before (Mean θ = 85.62° ± 28.9°, p < 0.001, Fig. 6A) and after (Mean θ = 84.09° ± 26.74°, p < 0.001, Fig. 6B) direction of sways were mostly oriented antero-posteriorly, and no significant differences were found for θ (F (1,82) = 0.05, p = 0.81) between before and after the manipulations.

### Subjective experiences

During the treatment, participants reported feeling their vertebrae moved under the therapist’s finger (36%), and that their tendons and muscles vibrated (58%) as “guitar strings” as the technique was respectively applied to the spinal segments, the ankles, and the feet. Participants also reported increased sweating (28%), and dizziness (42%). These sensations lasted after the manipulations up to two minutes for one patient but were shorter than a minute for the rest of the patients reporting such feedback. All side effects were gone at the end of the 5 min resting period. After the manipulations, patients reported less pain (72%), being freed in their motion (62%), and having less tension in their lower back (78%). These sensations lasted until the patient stepped out of the clinic.

## Discussion

The primary aim of this pilot study was to gain insight into whether proprioceptive manipulations modulate postural control in NSLBP patients when standing upright. We found that after the proprioceptive manipulations, COP speed and speed variance were significantly decreased, whereas sway mainly stayed oriented antero-posteriorly.

One could first point out that such decrease could be due to a learning effect as the experimental conditions were not randomized. Yet, this is probably unlikely. First, published postural data do not show statistical differences in COP outcomes during randomly repeated trials even when sensory stimulations are provided^33–35^. Second, published evidence also underlines the test–retest reliability of COP measurements is high^36–39^. Third, participants stood in a non-changing environment, eyes open on a flat hard surface. Therefore, the task did not represent any difficulty for a learning effect to account for the effect sizes we found for both speed and speed variance. Thus, given that (1) the performance measure is reliable, (2) the measured variables usually remain stable over time, and (3) the only alteration between the two measurements were the manipulations, we can hypothesize that, all other things being equal, the statistical differences herein could likely be due to the proprioceptive manipulations. Indeed, whatever caused these changes had to be sufficiently relevant to provide such effect sizes and nothing significant besides the manipulations occurred between the two measurements. Nonetheless, randomized placebo-controlled clinical trials will have to further investigate these effects.

Such results contrast with previous evidence showing that isolated ankles, feet, and lumbar manipulations, when the patients are lying down, do not seem to modulate COP speed^30–32^. In our study, we manipulated the lumbar spine, ankles, and feet within the same session. This is a limitation as we cannot untangle whether the decreased COP speed found herein could have resulted from spine, feet, or ankles manipulations alone or their combination. This distinction will have to be evaluated in future protocols.

Compared to other COP parameters, the COP speed shows the highest reliability and sensitivity^23,24^. It is also considered as a good postural biomarker as decreased COP speed reflects a more efficient postural control system overall^40^. Evidence shows that compared to healthy controls, NSLBP patients have increased AP body sway^41–43^. Moreover, chronic NSLBP patients often express higher COP speed measures^43–45^. Therefore, decreasing COP speed in acute NSLPB patients, as seen in our results, could be an interesting starting point in their rehabilitation process. This first point also raises the question of whether these proprioceptive techniques could help avoid translating NSLBP patients from acute to more chronic pain states. This could only be answered by future protocols, and we reckon that such research would be worth investigating.

Our results also show a significant decrease in the speed variance after we applied the proprioceptive manipulations. Published data underline a strong correlation between the speed variance and the activation of the overall postural muscles regardless of the amount of backward or forward body sway^25,26^. Increased speed variance has been related to increased body stiffening^26^. Thus, since speed variance was decreased after the proprioceptive manipulations, one could hypothesize that these manipulations could have impacted muscle activation by decreasing their tonic activity. Back pain patients responding to spinal manipulations show significant lumbar stiffness reduction^46^. Such a mechanism implies stiffening of the trunk and could potentially be linked to lumbar pain and altered postural control^15^. Interestingly, our results show not only a decrease in speed variance, but patients also self-reported less pain, less muscle tension, and a feeling of freedom in their capacity to move. The use of new methodological methods such as High-Density Electromyography (HDEMG) could help in providing answers on whether these proprioceptive techniques modulate muscles’ activation when patients experience low back pain^47^. Indeed, HDEMG has shown to be effective in giving a more precise representation of muscle behavior both spatially and temporally than more classical EMG recordings^47,48^.

Given that these proprioceptive manipulations are used in a clinical context, our secondary aim, in this pilot study, was to investigate how the patients subjectively felt. An important number of patients reported less pain after the manipulations. However, this point needs to be taken with a grain of salt. Considering the presumed effect on pain, it is worth noticing that our study was not intended to focus on pain levels. Pain is a very complex field of research and several uncontrolled parameters in our protocol could have inflated the responses in favor of decreased pain levels. For instance, placebo effects or a willingness to please the therapist cannot be underestimated. Yet, on the other hand, without being specifically questioned about their pain level, the patients freely and undirectedly self-reported their pain had been reduced. Therefore, future protocols could more precisely investigate and focus on the effects of the proprioceptive techniques on pain.

Taken together, five minutes after the proprioceptive manipulations were applied on patients standing up, our results show a better postural control. Such impact cannot be related to any biomechanical effect given (1) the low nature of the forces applied to the spinal processes, the ankles and the feet, (2) that no mechanical lever was used to manipulate the anatomical structures, (3) the fact that these techniques were not biomechanically oriented (i.e., always from right to left in this study) and (4) that these techniques were done upright when the antigravity muscle tone is not inhibited, thus considerably restraining the body parts movements. Therefore, our results are more likely due to other processes. Although only speculative at this point, there is a need to provide potential underlying mechanisms.

The patients reported feeling their body parts moving. Although we did not monitor for actual spinal and other movements, this is not likely due to biomechanical mechanisms given the nature of the techniques, the force applied, and the fact that muscle tone fighting against gravity would have avoided such movements. A more rational explanation could be that such perception of movement is generated by the brain being tricked by the somatosensory influx integration within the cortex. Apart from feeling spinal movements, the patients also reported feeling their body parts vibrating as the techniques were applied. Vibrations and kinesthetic cues applied to the skin and muscles have the capacity to induce spatial movement illusions^49–52^. Because these illusionary movements would not be generated by any self-produced motor command, they could be perceived as an externally generated balance perturbation^53^. Therefore, the techniques could be interpreted and integrated as exafferent information^1,54^ perturbating the ongoing postural control myogenic commands. Given that exafferent information is perceived as much stronger than reafferent cues^55^, the system could have been forced to find an appropriate motor solution to maintain proper balance. Indeed, postural control modulations, more specifically in the AP direction, are recorded immediately after vibrations are applied to muscles engaged in upright postural motor demands^56^. Altogether, the induced illusional movements tricking the brain into readjusting postural control online could have favored the emergence of new or alternative postural strategies.

We propose several different hypotheses that could explain such recalibration of postural strategies. First, so-called bimodal neurons encode both proprioceptive and vestibular information within the rostral Fastigial Nuclei (rFN) of the cerebellum^54^. Such cerebellar bimodal neurons explicitly respond to passive body motions^57^ and, in turn, play a major role in warranting accurate and proper posture and the maintenance of balance by sending strong neurological motor projections to the spinal cord^58^ and the vestibular nuclei^59^. Interestingly, such rFN neurons are also linked to vestibulo-autonomic function^58^, which could also explain why a subgroup of patients reported increased sweating and dizziness^60^. Illusionary passive perceived motions are often integrated and perceived as real physiological movements^52^. Furthermore, based on sensory inputs, the cerebellum is thought to play a crucial role in motor control by fine-tuning motor commands through internal models^61^. Therefore, by inducing a virtual perception of motion, the proprioceptive afferences could have updated the cerebellar models online, providing in return the motor commands needed to regulate the ongoing postural demands.

Second, the techniques were applied at the lumbar spine but also the ankles and the feet. When standing on a stable surface, NSLBP patients rely more on ankle proprioception than on their back muscles proprioception to maintain proper postural control^13,14^. This has been linked to a reorganization of the somatosensory cortex^14^. The result could therefore also emerge from a mechanism inverting the reweighting from the ankle to the lumbar spine, therefore normalizing the proprioception gains, and thus postural control.

Third, such strategies could also likely originate in cortical networks involving the motor cortex, given its important contribution to postural control^62^. Interestingly, low back pain patients with postural control deficits demonstrate modulations of cortical maps within their motor cortices^63^.

Fourth, patients with NSLBP have less low back proprioceptive acuity and awareness^12^, often associated with body schema disruptions^64^. Here again, skin and muscles vibrations and/or cues can also modulate the labile body schema representations^49^. Interestingly, tactile manipulations are positively reweighted when being upright while deficits emerge when lying down^65^.

Thus, the manipulations given in an orthograde posture could have optimized the integration of the proprioceptive cues. If so, they could have modulated and/or updated the cortical networks and maps in relation to embodiment mechanisms which are linked with how one’s body is perceived in space^66^. Moreover, such networks are thought to link together more specifically the intraparietal sulcus, the premotor cortex, the sensorimotor cortex, the extrastriate body area, and the temporoparietal cortex. All five cortical areas are known to bind visual, somatosensory, and vestibular signals^67–69^ all implicated in postural control^70^.

Motor control training is currently promoted to modulate sensorimotor neuroplasticity to improve LBP^15^. Such rehabilitation program considers targeting cortical networks integrating the primary motor cortex to fine-tune trunk muscle activations to help refine the motor control of the spine^15^. Whether the techniques in the present study reorganize cerebellar models and/or sensorimotor cortical maps, will have to be investigated in future protocols.

Rehabilitation strategies implementing motor control regimens have proved to be efficient in helping NSLBP patients^71,72^. Yet, training patients often required multiple sessions per day over days or even weeks to improve postural adjustments^73^. We only investigated the postural post-effects 5 min after we applied the techniques and unfortunately no follow-up of these patients was done. Therefore, we do not know the mid-and long-term effects of such manipulations which will have to be investigated in the future. Furthermore, although our results could be seen as encouraging, only randomized placebo-controlled clinical trials will provide more thorough answers as to which extent these proprioceptive techniques help the patients. Nonetheless, if future randomized placebo-controlled clinical trials were to show improved results through time, these techniques could help in gaining recovery time. If so, then perhaps these manipulations could avoid translating patients from acute lumbar to more chronic states which are ruining patients’ lives and economically burdening our societies^74,75^.

Future studies will also have to investigate the underlying mechanisms. Tactile and proprioceptive cues are more easily integrated in the upright posture than when lying down^65^. Thus, the singularity of the orthograde posture context could potentially open new therapeutic perspectives. If confirmed, with more thorough protocols, these results could also potentially be the beginning of a manual therapy paradigm shift combining simultaneously manual proprioceptive cues bound online with regulatory motor control commands. Given the postural effects, the techniques will also have to be tested on different populations. Indeed, they could provide an interesting solution for a broad range of patients such as the elderly, people experiencing dizziness and instabilities as well as mild traumatic brain-injured patients all experiencing postural and balance difficulties. Continuing investigating the effects of these proprioceptive techniques could, if proven effective, provide a cost-effective alternative not only for patients with musculoskeletal disorders^76^ but also for individuals with a broader range of dysfunctions.

## Conclusion

For the first time, to our knowledge, we investigated and showed improved withing-group postural stability after proprioceptive techniques were used. Contrary to more traditional manual therapies, such techniques were not applied on patients lying supine or prone, but in their physiological orthograde functional posture which requires not only simultaneous postural and motor commands but also online multisensory integration processes.
