# Author Correction: Proprioceptive manipulations in orthograde posture modulate postural control in low back pain patients: a pilot study

**DOI:** 10.1038/s41598-022-15933-w

**Published:** 2022-07-05

**Authors:** Nicolas Bouisset, Augustin Roland-Gosselin, Maurice Ouaknine, Jean Luc Safin

**Affiliations:** 1 Department of Kinesiology, Western University, London, ON N6A 3K7 Canada; 2 GRETM/Groupe de Recherche et d’Enseignement en Thérapie Manuelle, 03700 Bellerive-sur-Allier, France; 3 Université de Grenoble-Alpes, Laboratoire AGIM, 38700 La Tronche, France; 4 Institut de Formation en Masso-Kinésithérapie, 03200 Vichy, France

Correction to: *Scientific Reports* https://doi.org/10.1038/s41598-022-10701-2, published online 27 April 2022

The Competing interests section in the original version of this Article was incorrect.

"The authors declare no competing interests."

now reads:

"Both N.B. and A.R-G. declare no potential conflict of interest. M.O. has ties with Innovative Technology exploiting his patent regarding the Cyber-Sabots. J.-L.S. teaches the manual techniques evaluated in this work."

The original Article has been corrected.

